# Real-World Evidence on the Efficacy and Tolerability of Tramadol/Dexketoprofen (TRAM/DKP) Fixed-Dose Combination for the Management of Acute Non-surgical Pain in Asian Patients: A Multicentre Retrospective Case Series

**DOI:** 10.7759/cureus.41156

**Published:** 2023-06-29

**Authors:** Saran Tantavisut, Kok Yuen Ho, Edsel F Arandia, Sze Chung Cheng, Sarasate Eiamtanasate, Rahat Jarayabhand, Raymond Alvin J Kokseng, Jesse Jane L Paco, Gopinathan Raju, Prakrit Suwanpramote, Marvin Thepsoparn, Dinesh Nagrale

**Affiliations:** 1 Orthopaedics, Chulalongkorn University, Bangkok, THA; 2 Pain Management & Anaesthesiology, The Pain Clinic, Mount Alvernia Medical Centre, Singapore, SGP; 3 Orthopaedics, Philippine Orthopedic Center, Quezon City, PHL; 4 Orthopaedics and Traumatology, Core Health Centre, Kowloon, HKG; 5 Anaesthesiology, Faculty of Medicine Siriraj Hospital, Bangkok, THA; 6 Orthopaedics, Bhumibol Adulyadej Hospital, Bangkok, THA; 7 Orthopaedics, St. Luke's Medical Center - Global City, Taguig, PHL; 8 Anaesthesiology, Southern Philippines Medical Center, Davao del Sur, PHL; 9 Anaesthesiology and Pain Medicine, Pantai Hospital Kuala Lumpur, Kuala Lumpur, MYS; 10 Orthopaedics, Faculty of Medicine Ramathibodi Hospital, Bangkok, THA; 11 Pain Management Research Unit, Department of Anaesthesiology, Faculty of Medicine, King Chulalongkorn Memorial Hospital, Chulalongkorn University, Bangkok, THA; 12 General Practice, A. Menarini Asia-Pacific Holdings Pte. Ltd., Singapore, SGP

**Keywords:** fixed-dose combination, acute pain management, orthopedic pain management, tramadol dexketoprofen, asian, tramadol dexketoprofen drug combination, tolerability, treatment efficacy, acute non-surgical pain

## Abstract

Introduction: Multimodal analgesia is key in the effective management of acute pain. Previous clinical trials have demonstrated good results with the use of a fixed-dose combination (FDC) of tramadol 75 mg and dexketoprofen 25 mg (TRAM/DKP) in acute pain management. However, there is a dearth of real-world evidence on the efficacy and safety of this combination in the management of acute non-surgical pain, especially among Asian patients. The case series reported herein investigates the real-world experiences of physicians and Asian patients with the use of TRAM/DKP FDC in the management of acute non-surgical pain.

Methods: Data were collected retrospectively on 11 Asian patients across multiple hospitals who had received a short course of TRAM/DKP FDC for acute non-surgical orthopaedic and non-orthopaedic pain. Data on baseline characteristics, medical history, treatment regimen, clinical outcomes, and patient satisfaction were compiled and shared at a peer-to-peer expert meeting in October 2022.

Results: All patients experienced a reduction in pain intensity and were very satisfied with pain management, with a mean satisfaction score of 4.3/5. Five patients (range: 63-74 years) experienced mild adverse events, including nausea, vomiting, and dizziness, which resolved with no need for additional treatment in the majority of cases. No serious adverse events were recorded.

Conclusion: Asian patients with acute non-surgical orthopaedic and non-orthopaedic pain achieved good pain control with TRAM/DKP FDC. The regimen was well tolerated, and patients reported high levels of satisfaction with the outcomes, indicating that TRAM/DKP FDC is an effective choice for the control of acute non-surgical pain in Asian patients.

## Introduction

Effective assessment and management of pain is a critical component of patient care as acute pain is a common reason for emergency room visits [1,2]. Compared with chronic pain, there is a dearth of data on the prevalence of acute pain, especially within Asia. Evidence for the burden of acute non-surgical pain, which refers to pain of less than six months arising from a reason other than surgery [3], is largely unavailable. However, anecdotal evidence from healthcare professionals indicates that there is a considerable unmet need with regard to the management of acute non-surgical pain in Asia, similar to trends in the rest of the world [2]. Furthermore, the prevalence of inadequately managed cancer pain, a type of non-surgical pain, has been estimated to range between 27% and 79% in Asia [4]. Apart from the immediate discomfort caused by acute pain, ineffective management of acute pain can cause progression to chronic pain with resulting sequelae such as the decreased ability to carry out regular daily activities, reduced academic performance and/or productivity, increased risk of depression and suicidal thoughts, and reduced quality of life [5,6]. Thus, it is a priority to optimise the management of acute pain to prevent the transition to chronic pain.

The use of multimodal analgesia has gained widespread acceptance as an effective solution for the management of acute pain, including the treatment of acute non-surgical pain [7,8]. Considering the multiple pathogenic pathways from which pain originates, using two or more pharmacological agents to synergistically target multiple pain pathways simultaneously, achieving analgesic effects at a reduced risk of side effects makes multimodal analgesia appealing [9,10]. Clinicians now have the flexibility to choose combinations of non-steroidal anti-inflammatory drugs (NSAIDs), opioids, paracetamol (PCM), gabapentinoids, serotonin-norepinephrine reuptake inhibitors (SNRIs), and tricyclic antidepressants (TCAs) [7,10,11].

Fixed-dose combinations (FDCs) of analgesics provide the benefits of convenience, reduction in dose of individual compounds, and decreased pill burden [12], in addition to offering the advantages of multimodal analgesia. FDCs are easier to administer, thereby potentially improving patient adherence [13]. Several analgesic combinations have been developed for the management of acute pain, many of which are based on an opioid-nonopioid combination [12]. The combination of long-lasting tramadol hydrochloride 75 mg and rapid-acting dexketoprofen trometamol 25 mg (TRAM/DKP) in a fixed dose has demonstrated excellent results in clinical trials [14-17]. Dexketoprofen is an NSAID with analgesic and anti-inflammatory properties that act on the cyclo-oxygenase 1 and 2 pathways to alleviate pain [15]. Tramadol acts as a μ-opioid receptor agonist and an inhibitor of norepinephrine and serotonin reuptake [16]. Leveraging the unique benefits of the two classes of medication, studies have documented the superiority of TRAM/DKP FDC in the management of postoperative pain [15-17] and in the management of moderate-to-severe acute low back pain [14]. Furthermore, a 2019 Delphi study established the clinical use of TRAM/DKP FDC in the management of non-surgical pain settings such as dental, articular, traumatic, and low back pain [8].

While there is some evidence from clinical trials to guide the use of TRAM/DKP FDC in both surgical and non-surgical settings, there is a lack of real-world evidence on its efficacy and safety, particularly in the management of non-surgical acute pain in the Asian population. This case series report documents Asian patients’ experience within the context of efficacy and tolerability of TRAM/DKP FDC.

## Materials and methods

Physicians in Hong Kong, Malaysia, the Philippines, Singapore, and Thailand compiled case studies describing their experiences with TRAM/DKP FDC. The inclusion criteria for selected case studies were patients who had received a short course of tramadol (TRAM) 75 mg/dexketoprofen (DKP) 25 mg FDC (from The Menarini Group) for the treatment of acute moderate-to-severe non-surgical pain (moderate pain represented by scores of 5 to 6 on the numerical rating scale (NRS) and severe pain represented by NRS 7 to 10) [18]. Included patients were pre-evaluated for potential contraindications to both TRAM and NSAIDs, including renal impairment and gastrointestinal conditions. These cases were then discussed at a peer-to-peer expert meeting in October 2022. The primary objective of this expert meeting was to explore the role of TRAM/DKP FDC in the management of acute pain, elicit real-world evidence on its efficacy and tolerability among Asian patients, and ultimately optimise the management of acute non-surgical pain in Asia.

Baseline characteristics, including gender, age, and presence of comorbidities, were recorded. Patients were categorised into two groups based on whether they had non-surgical pain of orthopaedic or non-orthopaedic origin. Data on analgesic treatment regimen were noted and the clinical course following initiation of treatment was recorded. The endpoints for this study were pain intensity, patient satisfaction, and adverse events with TRAM/DKP FDC. The NRS and visual analogue scale (VAS) were used to assess pain intensity on a scale of 0 to 10. Patients’ level of satisfaction with response and tolerability of treatment was measured on a scale of 1 to 5, with 1 representing “not at all satisfied” and 5 representing “extremely satisfied”.

## Results

A total of 11 cases were included in this case series, with diagnoses ranging from pelvic fragility fracture and osteoarthritis to radiation-induced neuropathic pain of the sciatic nerve and severe dysmenorrhea from pelvic endometriosis. All patients experienced acute or acute-on-chronic moderate-to-severe pain requiring strong analgesics for pain control. Clinical details for each case are presented in Table 1 while the prevalence of adverse drug events is summarised in Table 2.

**Table 1:** Case studies of Asian patients who have received TRAM/DKP for the control of acute moderate-to-severe non-surgical pain NRS – numerical rating scale; VAS – visual analogue scale; TRAM/DKP – tramadol 75 mg + dexketoprofen 25 mg; FDC – fixed-drug combination; PCM – paracetamol; GP: general practitioner; TID – *ter in die*, three times daily; BID – two times daily; OD – once daily; PRN –* pro re nata*, as needed; PC – *post cibum*, after food; ON – *omni nocte*, at night; AM – *ante meridiem*, morning; HS – *hora somni*, at bedtime; IV – intravenous; PO –* per os*, by mouth; MMA – multimodal analgesia; CR – controlled release; IR: immediate release; RTC – return to clinic; ADL – activities of daily living; ED – emergency department; NSAIDs – non-steroidal inflammatory drugs.

Case	Pain type	Diagnosis	Case presentation	Pain management approach	Clinical outcomes	Satisfaction
Orthopaedic origin						
1	Left hip pain	Fragility fracture of the pelvis	74/F who fell on the left hip from standing height about five days earlier. Presented with severe pain (NRS 8/10 with motion, and 5/10 at rest). Previous treatment with tramadol with nausea/vomiting.	Day 1–5: TRAM/DKP FDC TID; PCM 500 mg six-hourly. Day 6–14: PCM 500 mg six-hourly PRN	Medication provided adequate pain relief, with pain significantly improved during the acute period, reduced from NRS 8/10 to 1–2/10.	4
2	Hip pain	Avascular necrosis of the hip	36/F who presented with acute exacerbation of a six-month history of hip pain. Imaging showed avascular necrosis of the hip and the patient was awaiting surgery.	Day 1–2: TRAM/DKP FDC TID. Day 3–5: TRAM/DKP FDC TID, PCM 1 g PO TID, fascia iliaca catheter insertion	The patient had surgery and fascia iliaca catheter insertion on day three, which remained in place for another two days. The pain was well controlled pre- and post-operatively with a reduction from NRS 7/10 to NRS 0–2/10 by day five on passive and active movement.	4
3	Mechanical back pain, radiation pain to legs	Back sprain injury, lumbar spine	63/F with an accidental back injury one week before presentation in the clinic. X-ray showed L4/L5 moderate degenerative disease but no fracture.	Day 1: TRAM/DKP FDC BID, physiotherapy. Day 2–5: TRAM/DKP FDC BID	Good pain relief with a reduction from VAS 7/10 to 1/10. Increased mobility by day five with the patient able to resume most ADL.	4
4	Left hip pain, chronic	Left hip osteoarthritis	72/F with a six-month history of progressive left hip pain, worse with start-up and prolonged weight-bearing/ambulation. Localisation of pain: left inguinal area with radiation along the anterolateral thigh. No antecedent trauma. Past medical history: S/P arthroscopic partial meniscectomy & debridement, right knee (four months prior).	Day 1: TRAM/DKP FDC TID, glucosamine sulphate 1500 mg OD, physical therapy (3x per week) starting from day 1. Day 2–5: TRAM/DKP FDC TID PRN, PCM 650 mg PO 6 to 8 hourly, glucosamine sulphate 1500 mg OD, physical therapy (3x per week)	Significantly reduced pain from VAS 8–9/10 to 0/10 at rest or 2–3/10 with activity. Increase in distance of ambulation and ability to engage in ADL; the patient was able to sleep better.	4
5	Severe right knee pain, new onset	Osteoarthritis-associated bone marrow lesion	66/F with new-onset acute moderate-to-severe right knee pain and swelling initially located at the posterior of the knee now localized to the medial joint line. The pain worsened three months ago. Denied history of trauma.	Day 1: TRAM/DKP FDC TID, glucosamine sulphate 500 mg TID, bisphosphonates, knee unloader brace, physical therapy (3x per week). Day 2–5: TRAM/DKP FDC TID, glucosamine sulphate 500 mg TID, knee unloader brace, physical therapy (3x per week). Day 6–11: dexketoprofen 25 mg TID	Acute flares on top of chronic inflammation, which caused a mixed type of moderate-to-severe pain were well managed with good pain relief (reduction from VAS 9/10 on activity to 3/10). Improved quality of life as the patient became more engaged in physiotherapy with increased mobility, got back to work and was able to continue with ADL.	4
6	Acute exacerbation of chronic cervical pain	Cervical spondylosis with disc herniation at C4/C5, C5/C6 with foraminal narrowing	55/M with a history of chronic neck pain secondary to cervical spondylosis; for which the patient takes tramadol/PCM FDC PRN. The patient fell while cycling and injured his left shoulder; presented in ER with no fracture on X-rays but mixed nociceptive-neuropathic pain at the lower neck and shoulder, which radiated to the left arm (VAS 5–7/10 at rest, 8–9/10 on movement). The patient was initially given IV tramadol and IV parecoxib in ED and discharged with pain medications but returned a few days later with intense pain. He was subsequently admitted and referred for pain management.	Day 1: TRAM/DKP FDC TID. Breakthrough: Oxycodone IR 5 mg, IV PCM 1 g. Day 2: TRAM/DKP FDC TID, metoclopramide PO PRN. Day 3: TRAM/DKP FDC BID, fluoroscopically-guided cervical epidural steroid injection with ropivacaine/dexamethasone. Day 4: TRAM/DKP FDC BID. Day 5: TRAM/DKP FDC BID; discharged with PCM and NSAIDs PRN	Pain reduced from NRS 8–9/10 on admission to 6–7/10 with MMA, then reduced further to 3–4/10 with interventional pain procedure and MMA on day three. The patient was very relieved, could finally sleep well and was able to move the neck freely without pain.	5
7	Right knee pain	Right knee osteoarthritis	42/F with a history of chronic low back pain with left radiculopathy due to L4/5 anterolisthesis and L4/5, L5/S1 exit foraminal and canal stenosis. The patient played competitive badminton and developed right knee pain and swelling after games.	Day 1–5: TRAM/DKP FDC TID, zolpidem CR 12.5 mg ON PRN	Reduction of pain from NRS 7/10 on ambulation and 5/10 at night which disturbed her sleep to NRS 2/10 at rest and on movement. The patient resumed ADL and sports; and had improved sleep quality.	5
8	Left shoulder pain	Cervical spondylosis with left radiculopathy	67/M who presented with left shoulder pain radiating to the left upper limb; no numbness or weakness. Received piroxicam and vitamin B supplements from GP for 1 week, but no improvement.	Day 1–5: TRAM/DKP FDC BID, pregabalin 75 mg PO BID	Significant reduction of pain from NRS 8/10 to 2/10 on movement and 1/10 at rest. The patient resumed ADL and had improved sleep quality.	4
Non-orthopaedic origin						
9	Right thigh pain	Radiation-induced neuropathic pain of sciatic nerve	52/F with a history of dedifferentiated liposarcoma (20 x 15 x 8 cm) 1+ year ago and had marginal excision of the liposarcoma with preservation of the sciatic nerve 3+ months ago. No history of significant pain during the peri-operative period. The patient subsequently received 36 doses of radiation therapy 3 weeks after surgery following which she developed progressive neuropathic pain in the right thigh 2 weeks prior.	Day 1–5: TRAM/DKP FDC TID, pregabalin 25 mg PO AM, pregabalin 75 mg PO HS, PCM 500 mg PO PRN four-hourly. Day 6–14: Pregabalin 25 mg PO AM, pregabalin 75 mg PO HS, PCM 500 mg PO PRN four-hourly	Significant pain relief with a reduction from NRS 6–8/10 to 3/10 on TRAM/DKP FDC before pregabalin reached a steady state. The patient could work and engagement in ADL was near-normal.	4
10	Acute pelvic pain from severe dysmenorrhea	Severe pelvic endometriosis with severe dysmenorrhea	32/F with a three-year history of severe pelvic endometriosis. Had three laparoscopic surgeries and used PCM, tramadol, pregabalin, and nortriptyline for the control of moderate chronic pelvic pain. The patient experiences severe dysmenorrhea every month with little relief from etoricoxib, PCM, and tramadol. The current episode of dysmenorrhea had left her unable to work and sleep and in a depressed mood.	Day 1 onwards: TRAM/DKP FDC TID, PCM 500 mg PO TID (for three to five days monthly)	Dysmenorrhea significantly reduced from NRS 8–9/10 to 4–5/10. The patient was able to get back to work during dysmenorrhea episodes with a better mood and improved sleep quality.	4
11	Left mandibular and left leg and knee pain	Diffuse large B-cell non-Hodgkin’s lymphoma (stage IV)	35/F with left mandibular, leg and knee pain that started three weeks prior. The pain was initially mild-to-moderate in intensity, aggravated by active movement and did not require medications until a day prior when it became so severe that she was unable to walk.	Day 1–3: TRAM/DKP FDC TID RTC. Day 4–7: dexketoprofen 25 mg TID	Significant improvement in pain from NRS 9/10 on day one to NRS 0/10 on day three. The patient was extremely satisfied with pain control; and able to go for further evaluation and treatment without pain and discomfort.	5

**Table 2:** Safety and tolerability outcomes reported in the case studies AEs – adverse events; PRN – *pro re nata*, as needed.

Case	Presentation	Diagnosis	Adverse events (AEs)
1	Left hip pain, five days	Fragility fracture of the pelvis	Minor symptoms of nausea, vomiting, and dizziness post-acute phase
2	Hip pain, six months	Avascular necrosis of the hip	Nausea (day one)
3	Mechanical back pain, radiation pain to legs	Back sprain injury, lumbar spine	No AEs observed
4	Left hip pain, chronic	Left hip osteoarthritis	Episodes of nausea & dizziness on day one (dosage adjusted to one tablet every 12 hours instead)
5	Severe right knee pain, new onset, three months	Osteoarthritis-associated bone marrow lesion	No AEs observed
6	Acute exacerbation of chronic cervical pain	Cervical spondylosis with disc herniation at C4/C5, C5/C6 with foraminal narrowing	Nausea (managed with PRN metoclopramide)
7	Right knee pain	Right knee osteoarthritis	No AEs observed
8	Left shoulder pain	Cervical spondylosis with left radiculopathy	No AEs observed
9	Right thigh pain, two weeks	Radiation-induced neuropathic pain of sciatic nerve	No AEs observed
10	Acute pelvic pain from severe dysmenorrhea	Severe pelvic endometriosis with severe dysmenorrhea	Mild nausea on day two, which improved later without treatment
11	Left mandibular and left leg and knee pain, three weeks	Diffuse large B-cell non-Hodgkin’s lymphoma (stage IV)	No AEs observed

Orthopaedic non-surgical pain

Case 1

Case 1 was a 74-year-old female presenting with acute severe left hip pain experienced since falling five days prior. Pain NRS was 8/10 on movement and 5/10 at rest. Previous treatment with tramadol provided minimal improvement. She was prescribed TRAM/DKP FDC three times daily (TID) alongside PCM 500 mg every six hours (QID) for five days. Thereafter, she was adjusted to PCM 500 mg QID as needed (PRN) until day 14. The patient was very satisfied, with pain NRS 1-2/10.

Case 2

Case 2 describes a 36-year-old female presenting with an acute exacerbation of hip pain that lasted six months. She had a history of systemic lupus erythematosus and chronic steroid use. Imaging during the current hospital visit showed features suggestive of avascular necrosis of the hip, and the patient was scheduled for surgery. For non-surgical pain management, the patient was commenced on TRAM/DKP FDC TID for five days. On day three, the patient had surgery and fascia iliaca catheter insertion, which remained in place for another two days. Also on day 3, the patient was prescribed PCM 1 g TID for three days. There was good pain relief both prior to and after the insertion of the fascia iliaca catheter with pain intensity reducing from NRS 7/10 at presentation to 0-2/10 by day five. The patient was very satisfied with pain management.

Case 3

Case 3 was a 63-year-old female who had recently suffered a traumatic injury to her lower back one week before presenting to the clinic. She complained of acute back pain, which radiated to her legs. On X-ray, there were features suggestive of L4/L5 moderate degenerative disc disease but no fractures. She was subsequently diagnosed with a back sprain of the lumbar spine. She was prescribed TRAM/DKP FDC twice daily (BID) and physiotherapy on day one. She continued with TRAM/DKP FDC BID for another four days and pain improved significantly from NRS 7/10 to 1/10. She was also able to increase mobility and resume most of her regular daily activities.

Case 4

Case 4 describes a 72-year-old female who presented with acute-on-chronic progressive pain in the left hip, which got worse with standing up, prolonged weight-bearing, and movement. The pain was most significant in the left inguinal area and radiated downwards along the anterolateral thigh. Prior to the presentation, she had arthroscopic partial meniscectomy and debridement in the right knee about four months ago. She was also hypertensive and obese (body mass index of 32 kg/m2), and X-rays showed signs of chronic osteoarthritis of the left hip. The patient was started on TRAM/DKP FDC TID and glucosamine sulphate 1500 mg once daily (OD) on day one. From day two to day five, she received TRAM/DKP FDC TID PRN, PCM 650 mg orally every six to eight hours, and continued glucosamine sulphate 1500 mg OD. She also did physical therapy three times during the week. By the end of treatment, she experienced a significant reduction in pain from VAS 8-9/10 to 0/10 at rest and 2-3/10 with some activity. She also improved ambulation, slept better, and could engage in regular daily activities.

Case 5

Case 5 was a 66-year-old female with new onset acute moderate-to-severe right knee pain that was initially felt behind the knee but subsequently localised to the medial joint line. The pain worsened three months prior to her hospital visit and there was no history of trauma. Imaging showed features indicative of osteoarthritis associated with bone marrow lesions. The patient was prescribed a short course of TRAM/DKP FDC TID for five days, after which she was switched to dexketoprofen 25 mg TID for another week. She also received glucosamine sulphate 500 mg TID for the first five days and bisphosphonates on the first day. She undertook physical therapy three times a week and used a knee unloader brace. By the end of week one, daily pain had reduced from VAS 9/10 on activity and rest to 3/10, and she was able to engage maximally in physical therapy, increase mobility, return to work, and continue regular daily activities.

Case 6

Case 6 describes a 55-year-old male who fell while cycling and injured his left shoulder. He presented to the emergency department (ED) with acute mixed nociceptive-neuropathic pain in the lower neck and left shoulder, which radiated to the left arm. He had a previous history of chronic neck pain secondary to cervical spondylosis for which he took tramadol/PCM FDC PRN. At the ED, X-rays showed no fractures and pain was VAS 5-7/10 at rest and 8-9/10 with movement. He was given IV tramadol and IV parecoxib and discharged with oral pain medications. However, he returned a few days later with intense pain and was admitted and referred for specialist pain management.

The patient complained of neck pain with radiation to the left arm and thumb, and tingling/pin prick-like sensations along the C5/C6 dermatome. Magnetic resonance imaging (MRI) showed cervical spondylosis with disc herniation at C4/C5 and C5/C6 with foraminal narrowing. He was prescribed TRAM/DKP FDC TID, oxycodone 5 mg PRN, and IV PCM 1 g TID. On day 3, TRAM/DKP FDC was reduced to twice daily and IV PCM was changed to oral PCM. He also underwent a fluoroscopically-guided cervical epidural steroid injection of ropivacaine 2% with dexamethasone 8 mg (day three). Pain first improved to NRS 6-7/10 with only TRAM/DKP FDC by day 2 vs. 8-9/10 on admission, after which it improved further to NRS 3-4/10. He continued with TRAM/DKP FDC BID on days four and five without any need for rescue oxycodone and was subsequently discharged with PCM PRN and NSAIDs. He reported sleeping well and was able to move his neck freely without pain.

Case 7

Case 7 was a 42-year-old female with a history of chronic low back pain and left radiculopathy due to L4/L5 anterolisthesis and L4/L5, L5/S1 exit foraminal and canal stenosis. She played competitive badminton and recently noted right knee pain and swelling after her games. The pain was 7/10 on ambulation and 5/10 at night, which interfered with her sleep. Examination and imaging showed features suggestive of osteoarthritis of the right knee. She was subsequently started on TRAM/DKP FDC TID and zolpidem 12.5 mg every night or as needed for five days. Pain reduced significantly to NRS 2/10 at rest and on movement, and her sleep improved. She also resumed regular daily activities and sports.

Case 8

Case 8 describes a 67-year-old male who presented with acute left shoulder pain radiating to his left upper limb. There was neither numbness nor weakness in the upper limbs. He had previously received piroxicam and vitamin B supplements from his general practitioner for approximately one week with no significant improvement in symptoms. He was switched to TRAM/DKP FDC BID and pregabalin 75 mg BID for five days. By day five, the pain had reduced to NRS 1/10 at rest and 2/10 with movement. He also resumed regular daily activities and could sleep better.

Non-orthopaedic non-surgical pain

Case 9

Case 9 was a 52-year-old female who had experienced right thigh pain for two weeks. One year previously, she was diagnosed with dedifferentiated liposarcoma (20 x 15 x 8 cm in size), which was removed about three months prior via marginal excision of the tumour with preservation of the sciatic nerve. There was no significant pain during the peri-operative period. Three weeks after surgery, she received 36 doses of radiation therapy and subsequently developed progressive right thigh pain (NRS 6-8/10) due to radiation-induced sciatic nerve neuropathy. She was started on 25 mg of oral pregabalin in the morning and 75 mg at bedtime, TRAM/DKP FDC TID, and oral PCM 500 mg every four hours PRN until day five. On day six, she was continued on pregabalin and PCM until day 14. The patient experienced significant pain relief with NRS decreasing from 6-8/10 to 3/10. She could also work and engage in regular daily activities.

Case 10

Case 10 was a 32-year-old woman with a previous history of severe pelvic endometriosis for three years and three laparoscopic surgeries. She had also been experiencing moderate chronic pelvic pain that was controlled with a combination of PCM, tramadol, pregabalin, and nortriptyline. She also experienced severe dysmenorrhea every month (NRS 8-9/10) with minimal relief with etoricoxib and tramadol. The current episode of dysmenorrhea had left her with severe pain, an inability to work or sleep well, and a depressed mood. She was prescribed a short course of TRAM/DKP FDC TID and PCM 500 mg orally TID. The dysmenorrhea subsequently improved with pain reducing from NRS 8-9/10 to 4-5/10, and the patient was able to return to work with a better mood and improved sleep quality.

Case 11

Case 11 was a 35-year-old female who presented with severe pain in the left knee and leg and left-sided mandibular pain that started three weeks prior to consultation. The pain was initially mild-to-moderate in intensity, aggravated on active movement, and did not require medications until a day prior to consultation when pain in the left knee became so severe that she could not walk (NRS 9/10). There was no history of fever or trauma. X-rays, computed tomography, and subsequent biopsy showed features consistent with diffuse large B-cell non-Hodgkin's lymphoma with extension to the femur, mandible, and ovaries. She was prescribed TRAM/DKP FDC TID for three days, after which she changed to dexketoprofen 25 mg TID for another four days. Excellent pain control was achieved by day three with NRS 0/10 at rest and minimal pain on active movement. The patient was able to proceed for further evaluation and treatment for the lymphoma without any pain or discomfort.

Tolerability

Among the 11 cases, only five reported nausea from the use of TRAM/DKP FDC. One patient required the use of metoclopramide PRN and another had the TRAM/DKP FDC dosage adjusted from TID to BID. In addition to nausea, dizziness and vomiting were observed in two and one patient, respectively. No serious adverse events were recorded.

## Discussion

This case series report documents the experience of using TRAM/DKP FDC for the management of acute non-surgical pain in 11 Asian patients. All patients had significant pain reduction with TRAM/DKP FDC. The average satisfaction scores for those with orthopaedic and non-orthopaedic type acute non-surgical pain were 4.25 and 4.33, respectively. Only five patients experienced mild adverse events, including nausea, vomiting, and dizziness. While physicians generally accept the use of TRAM/DKP FDC in elderly patients, this case series further lends credence to the tolerability of the combination in both younger and older Asian patients. Five of the 11 cases presented were older than 60 years (range: 63-74 years) and only three experienced mild nausea, vomiting, and dizziness, which resolved with little or no treatment.

The comprehensive results obtained from these cases provide evidence for the efficacy and tolerability of TRAM/DKP FDC in the management of acute non-surgical orthopaedic and non-orthopaedic pain. As previous clinical trials have demonstrated, there is a significant role for TRAM/DKP FDC in effectively managing acute moderate-to-severe pain [2,19]. Since the approval of TRAM/DKP FDC in Europe in 2016 and its introduction to the Asia-Pacific region in 2018 [8,20], anecdotal evidence from healthcare professionals indicates that many patients have benefited from TRAM/DKP FDC. A recent real-world study reported good efficacy and tolerability among Asian patients who received TRAM/DKP FDC for post-operative pain [20]. The acute non-surgical pain cases reported here add to this evidence, thus further establishing the important role of TRAM/DKP FDC in overall pain management. Targeting both central and peripheral sites in the pain pathway through synergistic and complementary mechanisms of action achieves better pain relief [2,8,13]. Within the context of acute non-surgical pain, the rapid onset of action of dexketoprofen complements the longer-lasting effect of tramadol to deliver a faster and prolonged analgesic effect [13]. This makes it possible for patients to recover rapidly and return to social activity and work.

Dexketoprofen has an anti-inflammatory effect, while tramadol acts on μ-opioid receptors and inhibits the reuptake of serotonin and norepinephrine in the descending pain pathway. It is thus effective in managing mixed nociceptive and neuropathic pain often seen in acute non-surgical pain [8,13]. Case 6 is a typical example of mixed nociceptive and neuropathic pain often seen in clinical settings. The occurrence of inflammation in response to recent trauma alongside the underlying neuropathic component from cervical spondylosis made a strong case for the use of TRAM/DKP FDC, which led to a significant reduction in the patient’s pain intensity. Similarly, there is documented evidence of the role of TRAM/DKP FDC in patients with acute exacerbations of low back pain, such as case 3, who might benefit from the central, peripheral, and anti-inflammatory actions of the combination [21]. However, despite the strong role of TRAM/DKP FDC in managing mixed pain, there may be a need to consider a combination with pregabalin during the short course of TRAM/DKP FDC before continuing with pregabalin for the long term. Gabapentinoids are first-line therapy in the management of neuropathic pain [9] and they are particularly relevant in patients who cannot continue with TRAM/DKP FDC beyond five days and/or have chronic neuropathic pain [10]. Also, due to the delayed onset of action of pregabalin, TRAM/DKP FDC can provide good initial pain control until pregabalin takes effect [22]. This points to the need for a combination of pregabalin and TRAM/DKP FDC in patients experiencing non-surgical pain with a strong neuropathic component.

Most patients in this study received the standard dose of TRAM/DKP FDC three times daily over five days. However, there were slight differences for some of the patients in terms of the duration between doses. Cases 3 and 8 with acute mechanical back pain following trauma and acute left shoulder pain, respectively, were both prescribed with TRAM/DKP FDC twice daily. Both patients were elderly (63 and 67 years) and experienced significant pain relief while on this treatment regimen. In these two cases, adjuncts such as physical therapy for case 3 and administration of pregabalin in case 8 may have played a role. Nevertheless, the excellent pain relief seen in both cases may illustrate an option to prescribe TRAM/DKP FDC twice daily instead. This is in line with an Italian study that reported good pain relief in patients with acute low back pain who had received TRAM/DKP FDC every 12 hours over five days [14].

Limitations

The retrospective nature of this study limits the reliability and validity of the findings, and as such, the results should be interpreted with caution. Cases were selected in a non-random manner across multiple healthcare facilities and countries. The resulting data were presented at a peer-to-peer expert meeting, which became the basis of the results in this manuscript. It is important to note that some data were missing for certain patients due to the retrospective collection of data. Additionally, the use of different treatment protocols may have an impact on the findings, especially with regard to the use of additional analgesic medications in addition to TRAM/DKP. Furthermore, the assessment of pain pre- and post-treatment was not standardised with regard to the tools used and assessment intervals. These limitations can be best avoided by using a prospective randomised trial following a standardised protocol in future studies.

## Conclusions

This case series has provided real-world evidence for the efficacy and safety of a short course of TRAM/DKP FDC in the management of acute non-surgical pain. Similar to the results of clinical trials evaluating the efficacy of TRAM/DKP among non-Asian patients, Asian patients with acute non-surgical pain achieved good pain control following administration over a five-day period. TRAM/DKP FDC was well-tolerated, and patients reported high levels of satisfaction, indicating that TRAM/DKP FDC is an effective choice for the control of acute non-surgical pain in Asian patients.
